# The efficacy of non-pharmacological interventions for treating constipation symptoms in lung cancer patients: a systematic review and network meta-analysis

**DOI:** 10.3389/fonc.2025.1633167

**Published:** 2025-08-18

**Authors:** Mian Cai, Jie Yin, Xiwei Fang, Yanjing Li, Liuxin Hu, Yiyong Xu

**Affiliations:** ^1^ Nursing of Colloge, Jiangxi University of Chinese Medicine, Nanchang, Jiangxi, China; ^2^ College of Clinical Medicine, Jiangxi University of Chinese Medicine, Nanchang, Jiangxi, China

**Keywords:** lung cancer, constipation, non-pharmacological intervention, NMA (network meta-analysis), systematic review

## Abstract

**Objective:**

This study aims to comprehensively evaluate the efficacy of different non-pharmacological interventions in treating constipation symptoms in lung cancer patients through a network meta-analysis, providing evidence-based support for personalized treatment decisions in clinical practice.

**Methods:**

We retrieved randomized controlled trials (RCTs) from well-known databases and compared the efficacy of non-pharmacological interventions with traditional treatments or placebos in improving constipation symptoms in lung cancer patients. The search was conducted through January 2025. Two researchers independently screened the literature, extracted data, and assessed the quality of the studies using the Cochrane risk of bias tool. Traditional meta-analysis was performed using Stata 16.0 software. Network meta-analysis (NMA) was conducted using RStudio software to integrate the data and create a network diagram to display the comparisons between non-pharmacological interventions. The credibility of the evidence was assessed using the Confidence in Network Meta-Analysis (CINeMA) tool.

**Results:**

A total of 33 studies involving 3,471 participants were included in this study. The traditional meta-analysis revealed that non-pharmacological interventions effectively reduced the incidence of constipation and improved constipation symptom management in lung cancer patients during treatment. Additionally, these interventions positively impacted the time to first bowel movement and reduced negative emotions (such as anxiety) in hospitalized lung cancer patients with constipation. The network meta-analysis (31 studies, 3,287 participants) indicated that acupoint stimulation was the most effective non-pharmacological intervention for reducing constipation incidence in lung cancer patients during treatment. The combined use of two types of acupoint patches showed optimal efficacy in improving constipation symptoms. However, for overall therapeutic effectiveness, the combination of acupoint patch therapy and acupoint massage most effectively reduced constipation incidence and improved overall constipation symptom management in lung cancer patients during treatment.

**Conclusion:**

The combination of acupoint patch therapy and acupoint massage is recommended as the preferred clinical intervention for treating constipation symptoms in lung cancer patients.

**Systematic Review Registration:**

https://www.crd.york.ac.uk/prospero/, identifier CRD42025631567.

## 1 Introduction

As of 2020, new cases of lung cancer accounted for 11.4% of all global cancer cases, making it the second most prevalent malignancy in terms of incidence (1). Particularly in China, lung cancer is the leading malignancy in terms of both incidence and mortality among male patients (2). Currently, surgery, radiotherapy, and chemotherapy are the primary clinical treatments for lung cancer (3). Constipation is one of the most common symptoms during anticancer treatment, particularly in patients with stage III/IV lung cancer. Chemotherapy drugs such as carboplatin and cisplatin, which are commonly used in these patients, are known to cause side effects like nausea and vomiting. To alleviate or control these symptoms, antiemetic drugs, such as ondansetron, are often required. While these medications can manage nausea and vomiting by suppressing gastrointestinal motility, they can also significantly increase the risk of constipation (4). Research has demonstrated that lung cancer patients exhibit a significant reduction in the abundance of beneficial bacteria such as Bifidobacteria and Lactobacilli in their gut microbiota, while the proportion of opportunistic pathogens like Enterococci increases, which consequently leads to intestinal dysfunction (5). Severe constipation in lung cancer patients increases cardiopulmonary burden and may even precipitate cardiovascular and cerebrovascular accidents. Although constipation management is crucial for patient health, existing research predominantly focuses on pain and cough management (6, 7). In recent years, non-pharmacological interventions (NPIs) have demonstrated distinct advantages in constipation management for lung cancer patients. In this study, NPIs are defined as an integrated intervention system covering diet, exercise, behavior therapy and external treatment of traditional Chinese medicine (such as acupuncture and moxibustion, massage and other standardized traditional therapies), so as to build a more clinical representative intervention network. These interventions are characterized by high safety profiles, absence of drug dependency, and hepatorenal toxicity risks, making them suitable for long-term use. Furthermore, NPIs can simultaneously address symptom relief and constitutional adjustment while offering significant economic cost-effectiveness. However, systematic exploration of NPIs for constipation management remains lacking. Current research on NPIs still presents limitations. Existing studies predominantly involve short-term follow-up periods and lack evidence supporting long-term efficacy and safety, making it difficult to comprehensively evaluate the durability and potential impacts of NPIs on constipation symptoms in lung cancer patients. Therefore, this study employs network meta-analysis, a statistical method that integrates direct and indirect evidence, to compare the efficacy and safety of various NPIs comprehensively. Through this methodology, we can systematically evaluate the relative advantages of different NPIs in constipation management for lung cancer patients, thereby providing more scientific and comprehensive evidence for clinicians to develop individualized treatment protocols.

## 2 Methods

### 2.1 Registration

This study was conducted according to the PRISMA-NMA guidelines and registered with PROSPERO (ID: CRD42025631567).

### 2.2 Literature search and screening

#### 2.2.1 Search strategy

We searched journals published up to January 2025 in PubMed, Embase, The Cochrane Library, Web of Science, CINAHL, China National Knowledge Infrastructure (CNKI), Wanfang Database, and VIP Database. The search keywords primarily focused on “Lung Neoplasms,” “Lung Cancer,” “Constipation,” “Colonic Inertia,” and related terms. The final search formula was determined by searching subject headings, subheadings, and free text terms. (See the **Supplementary Materials** for the PubMed search formula.)

#### 2.2.2 Literature screening

The retrieved literature was imported into the NoteExpress reference management software for screening. Two researchers (CM and YJ) independently conducted literature screening and data extraction. If there were any disagreements, they were resolved through discussion between the two parties or with the assistance of a third party (XYY). The extracted content included author, publication date, age, sample size, intervention protocol for the experimental group, intervention duration, outcome measures, and effect size.

#### 2.2.3 Inclusion and exclusion criteria

Inclusion criteria: Study type: The included studies encompass double-arm or multi-arm randomized controlled trials.

(P) Participants: The research subjects must be adults over 18 years old who have been diagnosed with lung cancer and have symptoms of constipation. There are no restrictions on the type, staging, or severity of lung cancer and constipation.

(I) Non-pharmacological interventions (NPIs) are the primary treatment methods, including but not limited to:

Traditional Chinese Medicine (TCM) interventions: moxibustion, acupuncture, massage, acupoint application, ear acupressure, etc.

Behavioral interventions: biofeedback training, scheduled toilet routines, relaxation techniques, etc.

Dietary and lifestyle interventions: fiber-rich diets, hydration protocols, structured physical activity, etc.

Studies employing single or multiple complementary and alternative medicine (CAM) interventions will also be considered.

(C) Control group intervention measures: These include routine treatment, placebo, sham intervention, blank control, or other different non-pharmacological intervention measures.

(O) Outcome indicators: Endpoint assessments must include at least one outcome indicator related to constipation or constipation-related measures under NPIs, such as effectiveness rate, incidence rate, etc. Outcome measurement types: This study uses the incidence of constipation symptoms and the effectiveness rate of treatment at the end of treatment as the primary outcome indicators. The time to first bowel movement recorded during treatment is a secondary outcome indicator.

(S) Study type: Randomized controlled trials (RCTs).

Exclusion criteria: Abstracts, unpublished academic papers, editorials, clinical observations, case studies, cohort design studies, non-randomized trials, and case-control studies.

#### 2.2.4 Data extraction and quality assessment

Two researchers (CM and YJ) created a data extraction table based on the information needed for the study, including author, year, research subjects, gender, sample size, intervention measures, follow-up time, outcome indicators, quality assessment information, and other relevant content. When the information extracted by the two individuals was inconsistent, they first discussed it with each other to resolve the issue. If the problem could not be resolved, a final decision was made through discussion with or consultation of a third researcher (XYY). Two researchers (CM and YJ) used the standards from the “Cochrane Handbook for Systematic Reviews of Interventions” to conduct quality assessments of the included RCTs across seven aspects: generation of random sequence, allocation concealment, blinding of researchers and subjects, blinding of study endpoints, completeness of outcome data, selective reporting, and other biases (8). The assessment was independently completed by both researchers and cross-checked. In case of disagreement, a third researcher (XYY) was consulted to determine the final assessment results. Additionally, we used the CINeMA (Confidence in Network Meta-Analysis) web application to assess the credibility of evidence, categorizing the credibility of results into four levels: high, moderate, low, and very low (9).

### 2.3 Statistical analysis

#### 2.3.1 Traditional meta-analysis

This study used Stata 16.0 software for traditional meta-analyses on extracted dichotomous and continuous variables. For dichotomous variables, odds ratios (OR) and 95% confidence intervals (CI) were used as effect indicators, while for continuous variables, standardized mean differences (SMD) and 95% CI were used as effect indicators. Results from different studies were combined using a random effects model. Heterogeneity between studies was assessed using the I² statistic and its associated p-value. Specifically, I² values of 25%, 50%, and 75% represented low, moderate, and high statistical heterogeneity, respectively. If P > 0.1 and I² ≤ 50%, this indicated low heterogeneity between studies, and a fixed effects model was applied for analysis; if P < 0.1 or I² > 50%, this indicated significant heterogeneity between studies, and a random effects model was applied for analysis, followed by subgroup and sensitivity analyses to infer the source of heterogeneity.

#### 2.3.2 Network meta-analysis

Network meta-analysis involves splitting all included studies into combinable two-arm comparisons and entering them into RStudio software. Markov chain Monte Carlo simulation chains were used to conduct network meta-analysis on primary outcomes including incidence rates and effectiveness, with odds ratio (OR) and its 95% confidence interval (CI) as effect size indicators. The BUGSnet 1.1.1 package was used for Bayesian network meta-analysis and to draw network relationship diagrams, calling JAGS (Just Another Gibbs Sampler). JAGS’ generalized linear model was used to process the included study data. The Bayesian network meta-analysis was set with 50,000 burn-in iterations, 100,000 formal iterations, and 10,000 adaptations for Markov chain Monte Carlo (MCMC) chain simulation. The potential scale reduction factor (PSRF) was used to evaluate the convergence results of the model, with a PSRF of 1.00-1.05 suggesting satisfactory model convergence. Network relationship diagrams of outcome indicators were drawn. When closed loops were formed in the diagram, the node-splitting method was used for inconsistency testing, with P > 0.05 suggesting consistency between direct and indirect comparison results. In the generated network diagram, each node represented different interventions and controls, and lines connecting nodes represented direct comparisons between different interventions. The size of each node and the width of connecting lines were proportional to the number of studies. The efficacy of each indicator was ranked to obtain the surface under the cumulative ranking (SUCRA), and the probability ranking was plotted on the chart. SUCRA was expressed as a percentage; the higher the percentage, the more effective the intervention, with a zero value indicating the intervention was ineffective.

## 3 Result

### 3.1 Literature search results

**Figure 1:**
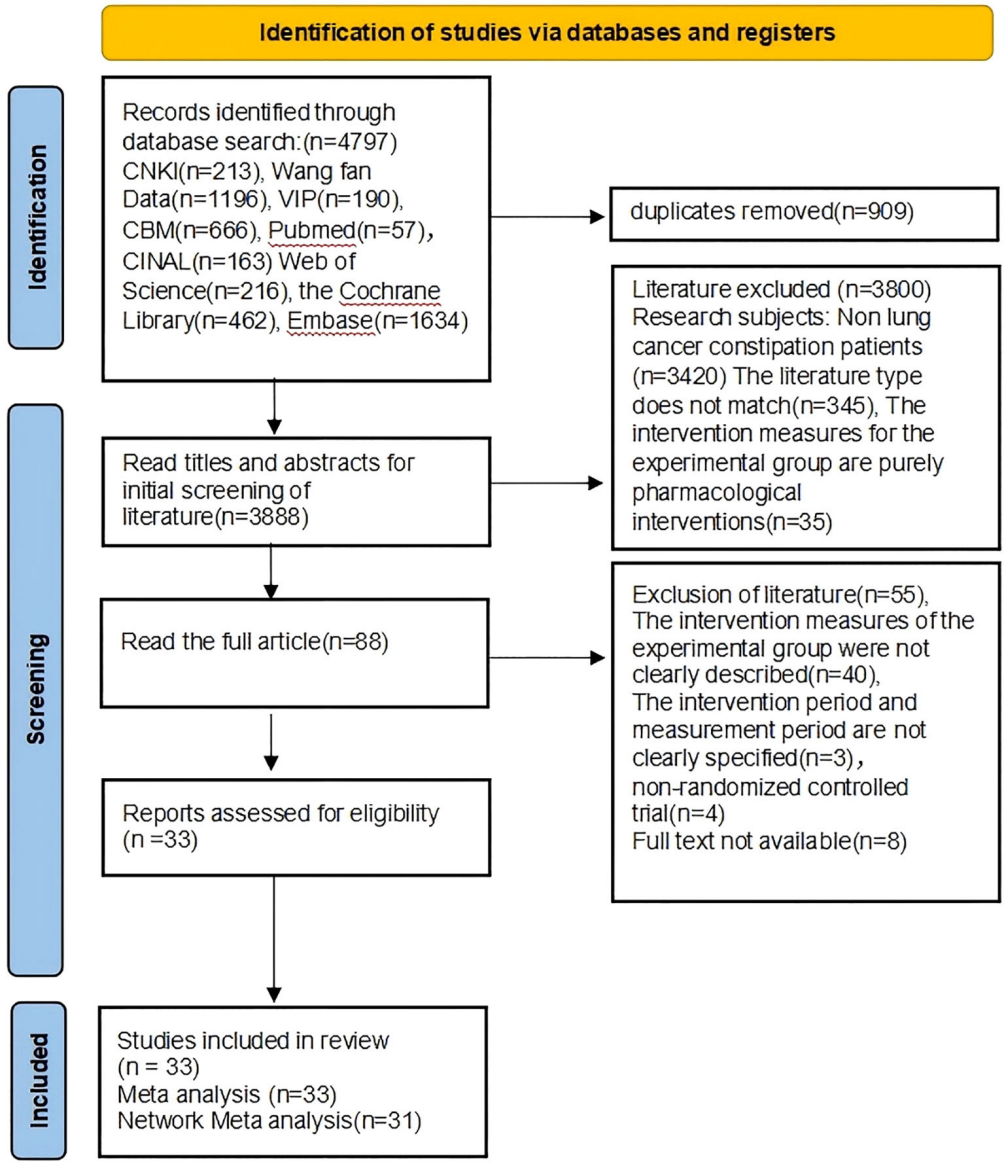
Literature screening process and results.

**Figure 1** shows the flowchart of PRISMA system overview. In the preliminary review stage, we conducted a comprehensive search of the database using retrieval methods and retrieved a total of 4797 relevant papers. We used NoteExpress software to remove 909 duplicate articles leaving 3888 articles. Subsequently, we conducted a first screening of the titles and abstracts, excluding 3800 studies, including 3420 articles unrelated to the research topic, 345 reviews and animal studies, and 35 studies involving drug interventions. For the remaining 88 relevant research articles, we conducted full-text reading for the second round of screening. Among them, full texts of 8 studies could not be found, and we attempted to contact the corresponding authors but were unable to obtain them. 40 studies did not clearly describe intervention measures, 3 studies did not describe intervention cycles and measurement times, and 4 studies were non-randomized controlled trials. Finally, 33 studies were included in the analysis, with all 33 studies included in the meta-analysis and 31 studies included in the network meta-analysis.

### 3.2 General characteristics

**Table 1** lists the characteristics of the included studies. Among the 33 included studies ( (10–42), a total of 3,471 patients met the analysis criteria. The interventions in these studies included exercise (including intestinal function training and therapeutic exercises), ear acupoint pressure, acupoint application, moxibustion, acupoint stimulation (including acupuncture and electroacupuncture), massage, probiotics, dietary interventions, and their combinations. Key characteristics were extracted, including sample size, intervention methods, treatment duration, and outcome measures.

**Table 1:** General situation table.

Author	Country/Language	Sample size		Intervention		Measure time	Outcome indicators
		t	c	t	c		
Li, Yan	China/English	174	167	CD	A	After the 3-day intervention is over	①⑤
Mao, T	China/English	61	61	K	A	Day 1, 14, 28	①
Wei, H	China/English	42	49	E	A	The first chemotherapy cycle ends, and the third chemotherapy cycle ends	①⑤
Chen Jingxiu	China/Chinese	40	40	I	B	On the first day and one week after surgery	①⑤
Song Yao	China/Chinese	25	25	IL	B	One day after surgery, 1 week after surgery	④
Qi Xiao	China/Chinese	40	40	D	A	7 days later	①
Qi Xiaofang	China/Chinese	28	24	DJ	A	On the day of chemotherapy and the third day	①
Xia Yanyan	China/Chinese	86	86	I	B	Day 1 and Day7	③
Li Danfeng	China/Chinese	53	53	F	D	Day 1 and Day 3	②③④
Lin Xiaoping	China/Chinese	60	60	GB	A	The first day of chemotherapy, on the 7th day of chemotherapy	①
Fan Guohua	China/Chinese	60;60;60	60	GH;G;H	A	7 days before and after the intervention	②
Chi Ying	China/Chinese	36	37	GD	A	On the 1st and 4th day after surgery	①
Wang Yuxian	China/Chinese	37	22	GD	A	Day 1, Day 2, and after the end of treatment	②
Ye Yuzhen	China/Chinese	50	50	H	A	Before and 3 weeks after the intervention	①③
Yang Yuqing	China/Chinese	31	31	H	A	On the first day of chemotherapy and the fourth day after the end of chemotherapy	①
Zhang Ying	China/Chinese	40	40	K	A	On the 3rd and 7th day of chemotherapy	①
Fengyinping	China/Chinese	20	20	D	A	Two weeks later	②
Jiang Luoyun	China/Chinese	25	25	HI	B	3 days after treatment	②④
Lu Jie	China/Chinese	36	36	DG	B	First defecation time and 10th day of treatment	②④
Liao Huilian	China/Chinese	100	100	D	B	Day 7.10.28	①
Xu Huiping	China/Chinese	44	44	G	B	Two weeks after the intervention	②
Yang Liu	China/Chinese	50	50	G	A	Day 2, Day 3, and 2 weeks later	②
Tian Weihua	China/Chinese	34	34	G	B	Two weeks later	②
Bian Xuemei	China/Chinese	93	93	G	A	Day 1 and Day 2	②
Yang Youfang	China/Chinese	60	60	G	A	After the end of treatment	①
Li Ran	China/Chinese	49	49	G	A	Day 3	①
Luo Mengmeng	China/Chinese	40	40	D	A	Day 10	①
Lou Danhua	China/Chinese	40	40	DK	B	After 1 week of intervention	②
Tong Yinxia	China/Chinese	53	56	D	B	Before and 2 weeks after intervention	②④
Xia Xueyun	China/Chinese	35	35	D	A	After 1 week of intervention	②
Ma Liang	China/Chinese	41	41	D	A	7 days later	②
Zhu Zhuwen	China/Chinese	60	60	D	B	After the intervention is completed	②
Hou Wei	China/Chinese	40	40	D	A	Day 1 and day 8	①

A: Routine care or placebo; B: Drug intervention; C: Ear pressing combined with acupoint application; D: Acupoint application; DG: Acupoint application combined with massage; DJ: Acupoint application combined with moxibustion; DK: Acupoint application combined with acupoint stimulation; E: Probiotic therapy; F: Multiple acupoint patches combined therapy; G: Massage; GB: Massage combined with medication; GH: Massage combined with dietary intervention; H: Dietary intervention; HI: Dietary intervention combined with exercise; I: Exercise; K: Acupoint stimulation; The English letters in the following pictures and tables represent the NPIs mentioned above. ① Incidence rate ② Effective rate ③ SAS ④ First defecation time ⑤ Adverse reactions

### 3.3 Quality assessment

**Figure 2:**
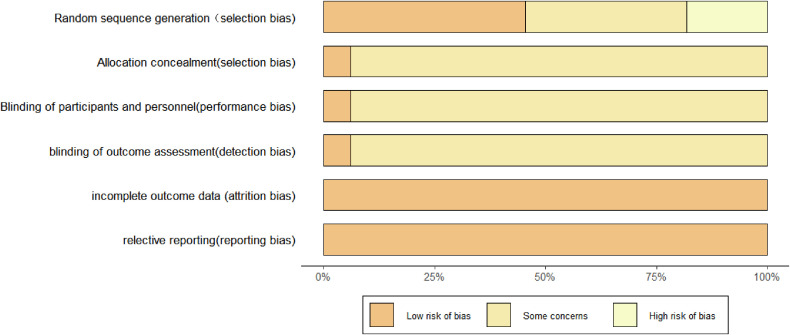
Evaluation of literature quality.

Using the Cochrane Collaboration’s RoB 2 tool, we assessed 33 included studies: 2 (6.1%) were judged to have low risk of bias, 25 (75.8%) had some concerns (primarily due to deviations from intended interventions or missing outcome data), and 6 (18.1%) were rated as high risk (especially the risk of bias in random processes). The overall risk-of-bias distribution is presented in **Figure 2**. For all outcome indicators, the overall certainty of evidence was relatively low. For the primary outcome indicators, we assessed the credibility of evidence in comparisons with the control group; all evidence was rated as moderate or low quality. In comparisons between different NPIs, most evidence was rated as low quality. Details can be found in the **Supplementary Figure 1**.

To evaluate how high-risk studies influenced the network estimates, we examined the distribution and impact of the 6 high-risk studies. These studies involved direct comparisons of exercise, acupoint application, massage combined with acupoint application, massage, and combined acupoint applications against control interventions, all contributing direct evidence to the network.

We conducted one-study-removed sensitivity analyses for the primary outcomes to assess the individual impact of each study, including the high-risk studies, on network estimates. Sensitivity analyses of 31 studies for primary outcomes (incidence rate and effectiveness rate) demonstrated that excluding any single study did not alter the pooled estimates (incidence rate: OR=0.16, 95%CI [0.11–0.24], P<0.01; effectiveness rate: OR=3.41, 95%CI [1.75–6.65], P<0.01). This finding indicates that the network estimates remained stable even when high-risk studies were individually removed, suggesting that the inclusion of studies with methodological concerns did not substantially bias the overall network meta-analysis results. The robustness of these findings supports the reliability of our primary conclusions, despite the presence of studies with high risk of bias in the network.

### 3.4 Conventional meta-analysis

#### 3.4.1 Incidence rate

**Figure 3:**
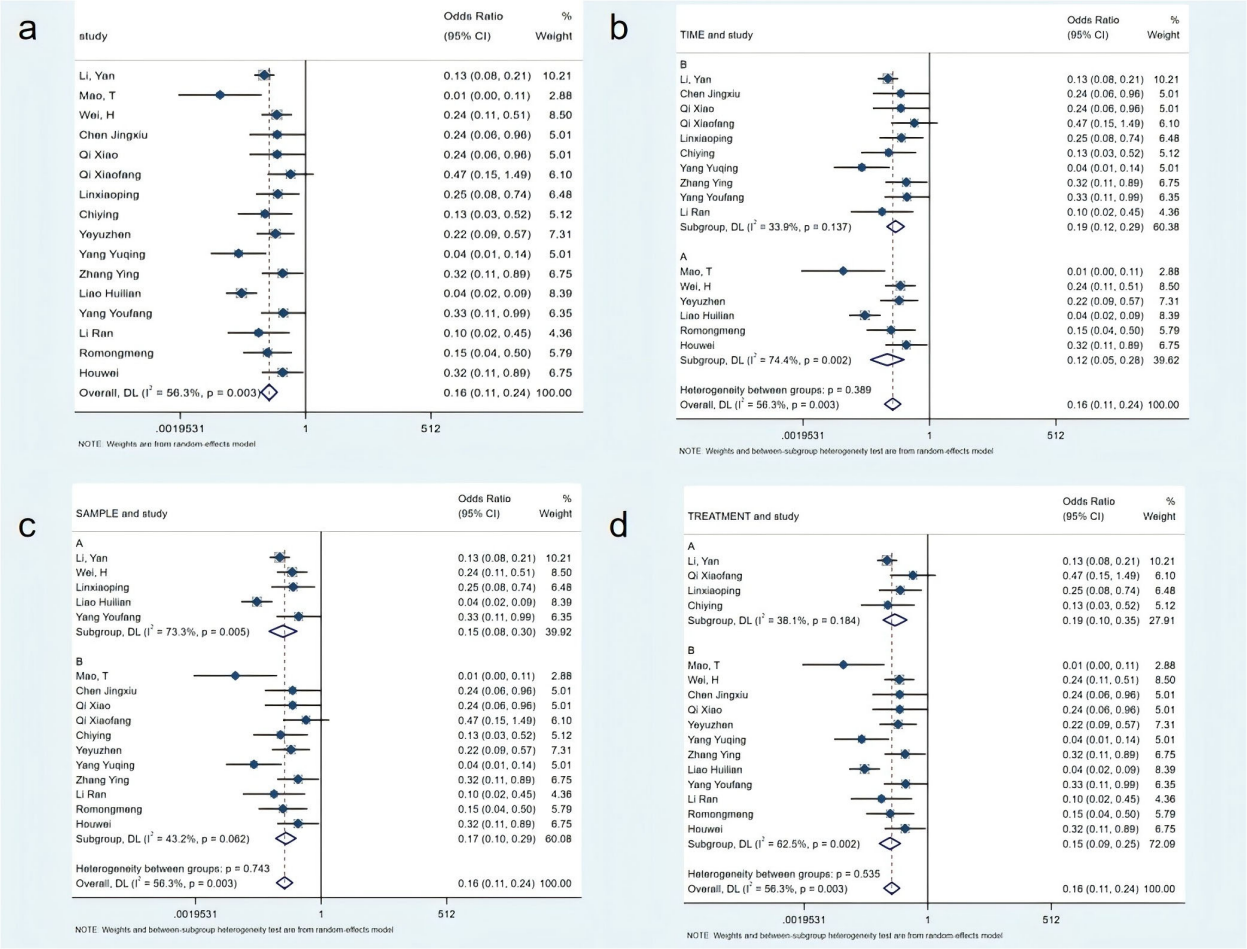
Meta-analysis of incidence rates. **(a)** Meta-analysis of incidence rate; **(b)** Subgroup analysis of incidence rate based on intervention time; **(c)** Subgroup analysis of incidence rate based on sample size; **(d)** Subgroup analysis of incidence rate by sample size.

16 studies with 1,779 participants evaluated the incidence rate of constipation symptoms after treatment. Meta-analysis showed that the incidence rate in the non-pharmacological intervention group was significantly reduced (OR=0.16; 95% CI [0.11, 0.24], P<0.01), with moderate heterogeneity (I²=56.3%). Subgroup analysis results showed that studies with intervention duration ≤7 days had moderate heterogeneity (I²=33.9%), while those with intervention duration >7 days had moderate-to-high heterogeneity (I²=74.4%). Regarding sample size, the subgroup with a total sample size ≥120 showed moderate-to-high heterogeneity (I²=73.3%), while the group with a sample size <120 showed moderate-to-low heterogeneity (I²=43.2%). I In terms of intervention types, the combined non-pharmacological intervention groups showed moderate-to-low heterogeneity (I²=38.1%). However, the single non-pharmacological intervention group showed moderate-to-high heterogeneity (I²=62.5%) (see **Figure 3**).

#### 3.4.2 Effective rate

**Figure 4:**
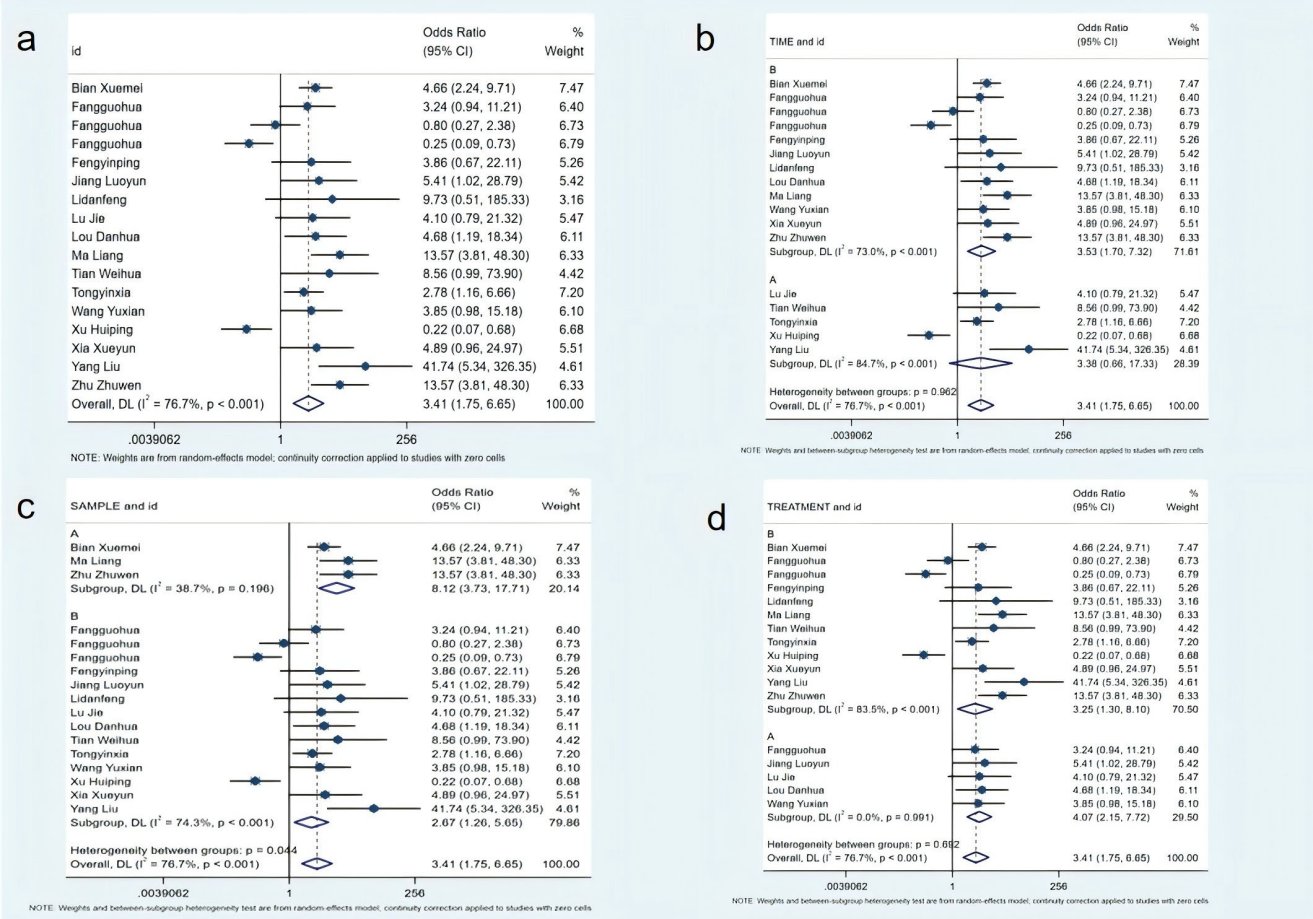
Meta-analysis of effective rate. **(a)** Meta-analysis of effective rate; **(b)** Subgroup analysis of adequate intervention time; **(c)** Subgroup analysis of adequate sample size; **(d)** Subgroup analysis of effective intervention measures.

A total of 15 studies, comprising 1,508 patients, were included in the evaluation of the effective rate of constipation symptom treatment. Meta-analysis showed that the non-pharmacological intervention group had a significantly higher effective rate (OR=3.41; 95% CI [1.75, 6.65], P<0.01). Subgroup analysis results showed that the group with intervention duration ≥7 days had moderate-to-high heterogeneity (I²=73%), while the group with intervention duration <7 days had extremely high heterogeneity (I²=84.7%). The group with a sample size ≥120 had moderate heterogeneity (I²=51%), while the group with a sample size <120 had moderate-to-high heterogeneity (I²=76.3%). Regarding intervention types, the combined non-pharmacological intervention groups had extremely low heterogeneity (I²=0), while the single intervention group had extremely high heterogeneity (I²=83.5%) (see **Figure 4**).

#### 3.4.3 Self-Rating Anxiety Scale

**Figure 5:**
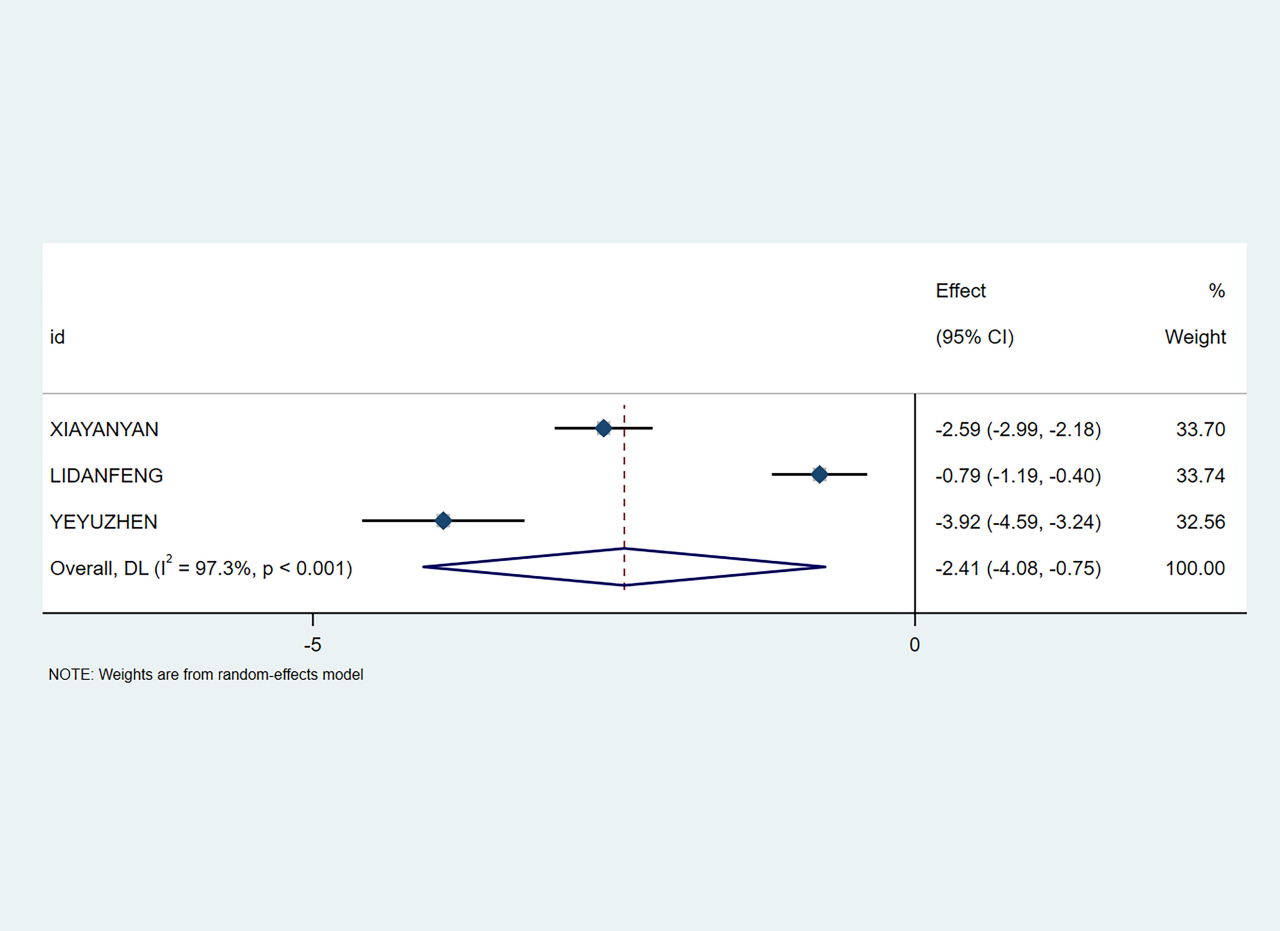
Meta-analysis of SAS.

A total of 3 studies involving 378 patients evaluated the Self-Rating Anxiety Scale (SAS) scores. Meta-analysis showed that the non-pharmacological intervention group effectively reduced patients’ post-intervention SAS scores (SMD=-2.41; 95% CI [-4.08, -0.75], P<0.01) (see **Figure 5**).

#### 3.4.4 Time to first bowel movement

**Figure 6:**
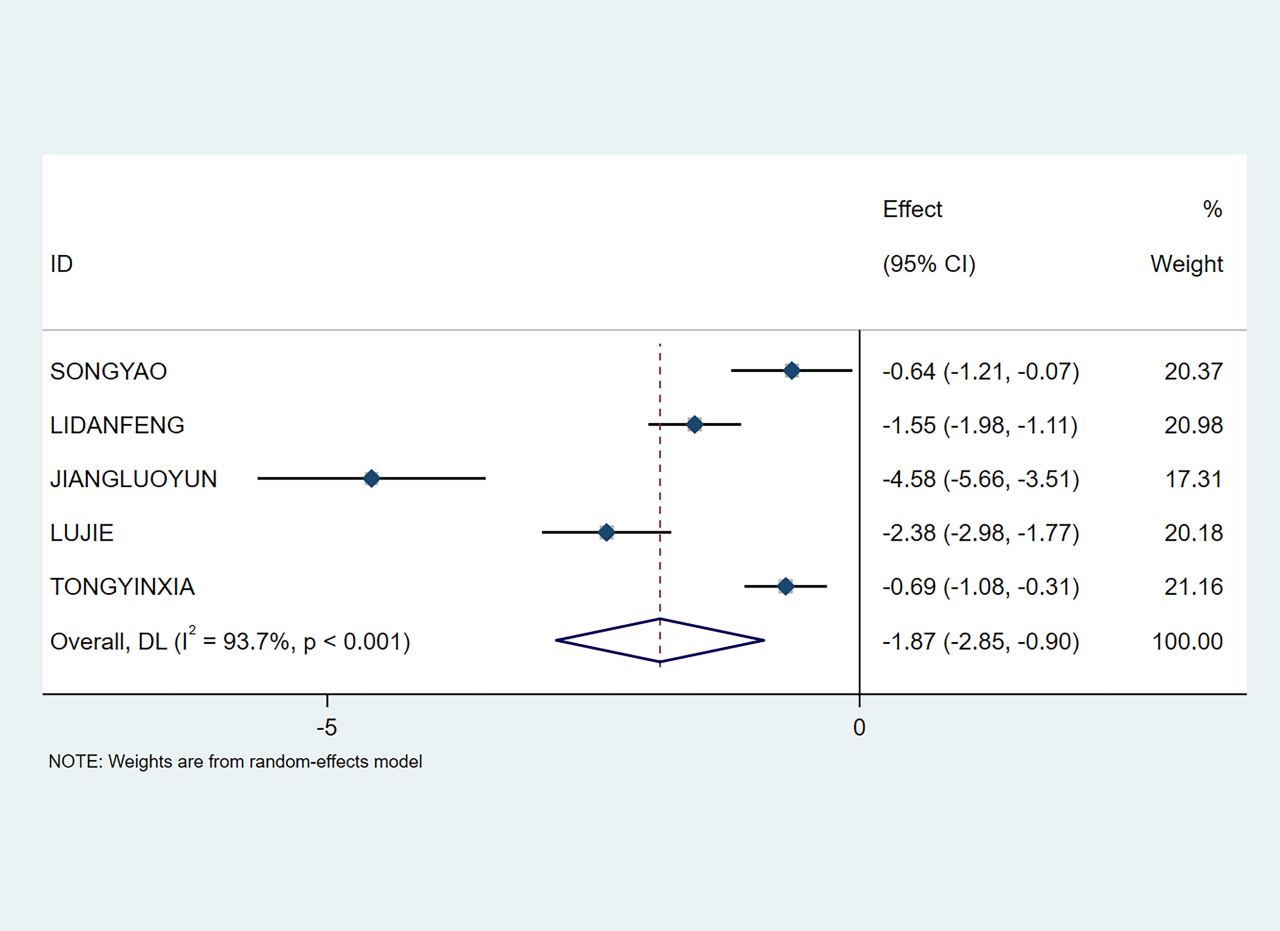
Meta-analysis of time to first bowel movement.

A total of 5 studies involving 387 participants reported the time to first bowel movement. Meta-analysis showed that NPIs could effectively shorten the time patients took for their first bowel movement (SMD=-1.87; 95% CI [-2.85, -0.9], P<0.01) (see **Figure 6**).

### 3.5 Network meta-analysis

**Figure 7:**
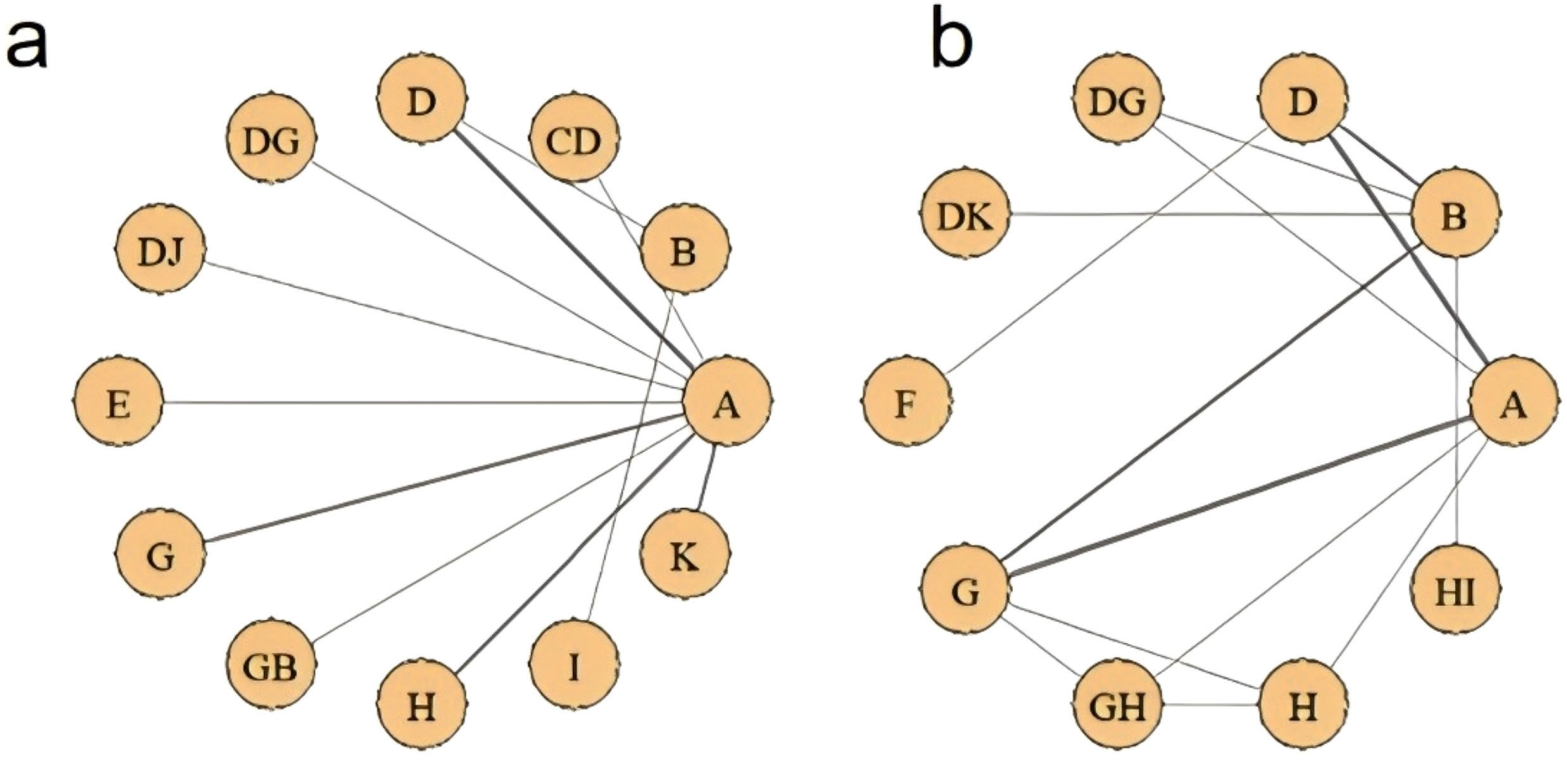
NMA. **(a)** NMA of incidence rate; **(b)** NMA of effective rate.

**Figure 7** presents the eligible comparison network diagram of different NPIs for constipation symptoms in lung cancer patients. 31 studies involving 3,827 participants evaluated the effects of various NPIs on constipation symptoms in lung cancer patients. All NPIs were directly compared with at least the control group.

**Figure 8:**
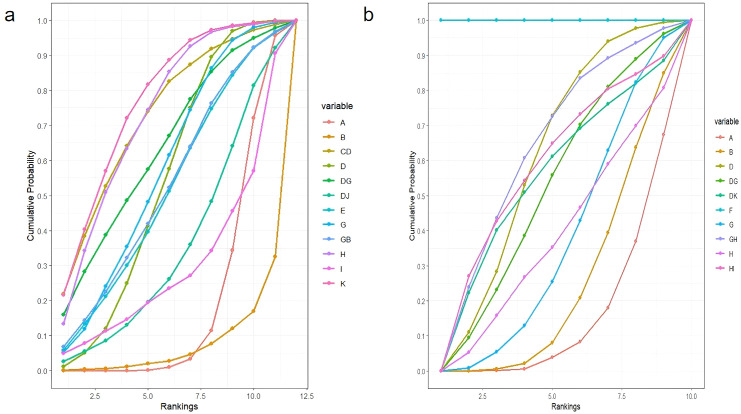
Ranking of SUCRA. **(a)** Ranking of SUCRA incidence rates; **(b)** Ranking of SUCRA effective rates.

Among NPIs for reducing the incidence of constipation symptoms after treatment in lung cancer patients, only one intervention showed a statistically significant reduction in the incidence of constipation symptoms after treatment compared to the routine control group: acupoint stimulation. Compared with the laxative control group, although various NPIs showed lower incidence rates of constipation symptoms after treatment in lung cancer patients, none of these differences were statistically significant (details shown in **Table 2**). According to the SUCRA results, for reducing the incidence of constipation symptoms after treatment in lung cancer patients, acupoint stimulation ranked highest with a SUCRA probability of 77%, followed by ear acupuncture combined with acupoint application ranking second with a SUCRA probability of 74%, and dietary intervention ranking third with a SUCRA probability of 73%. The remaining interventions ranked as follows: acupoint application combined with massage (SUCRA=70%), massage (SUCRA=60%), acupoint application (SUCRA=54%), probiotics (SUCRA=51%), massage combined with laxatives (SUCRA=50.0%), acupoint application combined with moxibustion (SUCRA=40%), exercise (SUCRA=28%), routine care group (SUCRA=19%), and laxative group (SUCRA=6%) (see **Figure 8a**).

**Table 2:** Incidence rate league table.

Intervention	A	B	CD	D	DG	DJ	E	G	GB	H	I
A	A	1.771 (-2.311, 5.641)	-2.543 (-6.093, 0.891)	-1.488 (-3.627, 0.633)	-2.137 (-5.576, 1.326)	-0.969 (-4.513, 2.94)	-1.358 (-5.144, 2.071)	-1.808 (-4.563, 0.833)	-1.523 (-4.979, 2.182)	-2.476 (-5.03, 0.151)	0.162 (-5.047, 5.97)
B	-1.771 (-5.641, 2.311)	B	-4.412 (-9.599, 0.829)	-3.25 (-6.704, 0.223)	-3.872 (-8.757, 1.481)	-2.636 (-8.198, 3.015)	-3.138 (-7.959, 2.021)	-3.665 (-8.044, 1.244)	-3.291 (-8.35, 2.057)	-4.238 (-8.742, 0.582)	-1.545 (-4.855, 2.506)
CD	2.543 (-0.891, 6.093)	4.412 (-0.829, 9.599)	CD	1.036 (-2.872, 5.372)	0.415 (-4.479, 5.792)	1.679 (-3.192, 7.19)	1.094 (-3.79, 6.744)	0.729 (-3.897, 5.049)	0.979 (-3.624, 6.714)	0.141 (-4.019, 4.756)	2.648 (-3.175, 9.424)
D	1.488 (-0.633, 3.627)	3.25 (-0.223, 6.704)	-1.036 (-5.372, 2.872)	D	-0.66 (-4.29, 3.338)	0.658 (-3.639, 5.176)	0.087 (-3.612, 4.169)	-0.344 (-3.709, 3.045)	0.014 (-4.001, 4.354)	-0.936 (-4.259, 2.241)	1.746 (-3.118, 6.983)
DG	2.137 (-1.326, 5.576)	3.872 (-1.481, 8.757)	-0.415 (-5.792, 4.479)	0.66 (-3.338, 4.29)	DG	1.19 (-3.699, 6.161)	0.769 (-3.939, 5.736)	0.329 (-4.289, 4.454)	0.485 (-4.577, 5.895)	-0.325 (-4.773, 4.072)	2.375 (-4.183, 8.789)
DJ	0.969 (-2.94, 4.513)	2.636 (-3.015, 8.198)	-1.679 (-7.19, 3.192)	-0.658 (-5.176, 3.639)	-1.19 (-6.161, 3.699)	DJ	-0.432 (-5.432, 4.076)	-0.946 (-5.577, 3.248)	-0.643 (-5.729, 4.582)	-1.49 (-6.378, 3.061)	1.084 (-5.389, 7.878)
E	1.358 (-2.071, 5.144)	3.138 (-2.021, 7.959)	-1.094 (-6.744, 3.79)	-0.087 (-4.169, 3.612)	-0.769 (-5.736, 3.939)	0.432 (-4.076, 5.432)	E	-0.407 (-4.722, 3.71)	-0.108 (-4.796, 4.751)	-1.089 (-5.113, 3.169)	1.468 (-4.575, 7.902)
G	1.808 (-0.833, 4.563)	3.665 (-1.244, 8.044)	-0.729 (-5.049, 3.897)	0.344 (-3.045, 3.709)	-0.329 (-4.454, 4.289)	0.946 (-3.248, 5.577)	0.407 (-3.71, 4.722)	G	0.276 (-4.178, 4.906)	-0.584 (-4.424, 3.374)	2.038 (-3.612, 8.161)
GB	1.523 (-2.182, 4.979)	3.291 (-2.057, 8.35)	-0.979 (-6.714, 3.624)	-0.014 (-4.354, 4.001)	-0.485 (-5.895, 4.577)	0.643 (-4.582, 5.729)	0.108 (-4.751, 4.796)	-0.276 (-4.906, 4.178)	GB	-0.853 (-5.48, 3.633)	1.689 (-5.02, 8.135)
H	2.476 (-0.151, 5.03)	4.238 (-0.582, 8.742)	-0.141 (-4.756, 4.019)	0.936 (-2.241, 4.259)	0.325 (-4.072, 4.773)	1.49 (-3.061, 6.378)	1.089 (-3.169, 5.113)	0.584 (-3.374, 4.424)	0.853 (-3.633, 5.48)	H	2.578 (-3.176, 8.68)
I	-0.162 (-5.97, 5.047)	1.545 (-2.506, 4.855)	-2.648 (-9.424, 3.175)	-1.746 (-6.983, 3.118)	-2.375 (-8.789, 4.183)	-1.084 (-7.878, 5.389)	-1.468 (-7.902, 4.575)	-2.038 (-8.161, 3.612)	-1.689 (-8.135, 5.02)	-2.578 (-8.68, 3.176)	I
K	2.621 (-0.062, 5.592)	4.378 (-0.362, 9.37)	0.179 (-4.703, 4.366)	1.13 (-2.357, 4.653)	0.536 (-3.896, 4.863)	1.711 (-2.639, 6.369)	1.269 (-2.801, 5.461)	0.835 (-3.074, 4.721)	1.13 (-3.16, 6.146)	0.198 (-3.765, 4.149)	2.753 (-2.998, 9.065)

Regarding improving the effectiveness rate of constipation symptom treatment in lung cancer patients, NPIs showed significantly higher effectiveness rates than the routine control group. However, only two interventions demonstrated statistically significant differences: single acupoint application and combined use of two types of acupoint applications (detailed information shown in **Table 3**). For improving the effectiveness rate in treating constipation symptoms in lung cancer patients, combined use of two types of acupoint patches ranked highest (SUCRA=100%), followed by massage combined with dietary intervention, ranking second (SUCRA=61.35%), and acupoint application, ranking third (SUCRA=61.33%). The remaining interventions ranked as follows: dietary intervention combined with exercise (SUCRA=60%), acupoint application combined with acupoint stimulation (SUCRA=57%), acupoint application combined with massage (SUCRA=50%), dietary intervention (SUCRA=36%), massage (SUCRA=35%), laxatives (SUCRA=24%), and routine care (SUCRA=14.0%) (see **Figure 8b**).

**Table 3:** Effective rate league table.

Intervention	A	B	D	DG	DK	F	G	GH	H	HI
A	A	0.461 (-1.894, 2.936)	2.245 (0.001, 4.142)	1.575 (-1.318, 4.721)	2.248 (-2.144, 6.828)	34.438 (13.594, 76.504)	0.88 (-1.047, 3.228)	2.367 (-1.162, 6.427)	0.995 (-2.562, 5.142)	2.471 (-2.294, 7.627)
B	-0.461 (-2.936, 1.894)	B	1.746 (-0.711, 3.96)	1.128 (-1.777, 4.466)	1.798 (-2.324, 5.62)	33.553 (12.461, 76.596)	0.446 (-1.821, 2.976)	1.864 (-1.866, 6.11)	0.517 (-3.251, 4.831)	2.021 (-2, 6.516)
D	-2.245 (-4.142, -0.001)	-1.746 (-3.96, 0.711)	D	-0.518 (-3.873, 2.847)	0.08 (-4.425, 4.83)	31.727 (10.958, 74.2)	-1.288 (-3.893, 1.474)	0.178 (-3.56, 4.873)	-1.24 (-5.146, 3.296)	0.146 (-4.445, 5.473)
DG	-1.575 (-4.721, 1.318)	-1.128 (-4.466, 1.777)	0.518 (-2.847, 3.873)	DG	0.571 (-4.426, 5.408)	32.392 (11.256, 75.193)	-0.764 (-3.823, 2.552)	0.645 (-3.916, 5.456)	-0.742 (-5.284, 4.443)	0.812 (-4.36, 6.409)
DK	-2.248 (-6.828, 2.144)	-1.798 (-5.62, 2.324)	-0.08 (-4.83, 4.425)	-0.571 (-5.408, 4.426)	DK	31.918 (10.493, 74.166)	-1.273 (-5.748, 3.502)	0.093 (-5.23, 5.958)	-1.316 (-6.992, 4.634)	0.172 (-5.42, 6.53)
F	-34.438 (-76.504, -13.594)	-33.553 (-76.596, -12.461)	-31.727 (-74.2, -10.958)	-32.392 (-75.193, -11.256)	-31.918 (-74.166, -10.493)	F	-33.28 (-76.087, -12.183)	-31.678 (-74.273, -10.758)	-33.04 (-75.68, -12.34)	-31.835 (-75.249, -10.517)
G	-0.88 (-3.228, 1.047)	-0.446 (-2.976, 1.821)	1.288 (-1.474, 3.893)	0.764 (-2.552, 3.823)	1.273 (-3.502, 5.748)	33.28 (12.183, 76.087)	G	1.486 (-2.107, 5.004)	-0.005 (-3.462, 3.633)	1.51 (-3.329, 6.87)
GH	-2.367 (-6.427, 1.162)	-1.864 (-6.11, 1.866)	-0.178 (-4.873, 3.56)	-0.645 (-5.456, 3.916)	-0.093 (-5.958, 5.23)	31.678 (10.758, 74.273)	-1.486 (-5.004, 2.107)	GH	-1.414 (-5.207, 2.694)	0.063 (-5.872, 5.949)
H	-0.995 (-5.142, 2.562)	-0.517 (-4.831, 3.251)	1.24 (-3.296, 5.146)	0.742 (-4.443, 5.284)	1.316 (-4.634, 6.992)	33.04 (12.34, 75.68)	0.005 (-3.633, 3.462)	1.414 (-2.694, 5.207)	H	1.433 (-4.703, 7.538)
HI	-2.471 (-7.627, 2.294)	-2.021 (-6.516, 2)	-0.146 (-5.473, 4.445)	-0.812 (-6.409, 4.36)	-0.172 (-6.53, 5.42)	31.835 (10.517, 75.249)	-1.51 (-6.87, 3.329)	-0.063 (-5.949, 5.872)	-1.433 (-7.538, 4.703)	HI

**Figure 9:**
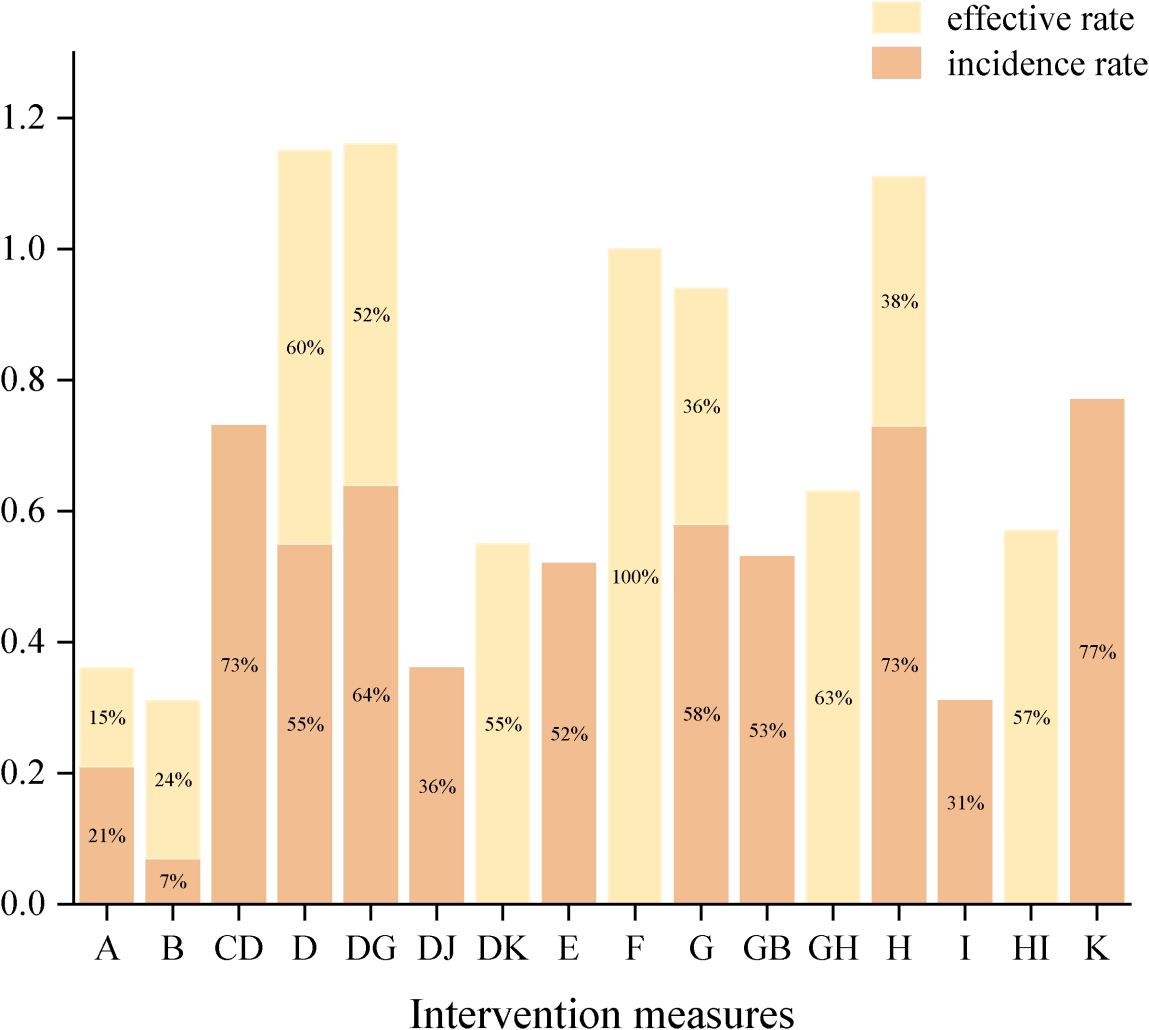
Comprehensive sorting chart.

Finally, based on SUCRA rankings across different outcome indicators, acupoint application combined with massage ranked highest overall in treating constipation symptoms in lung cancer patients (see **Figure 9**). It is important to note that these rankings represent treatment hierarchy based on probability estimates rather than definitive clinical superiority, and should be interpreted alongside the statistical significance of pairwise comparisons and clinical considerations.

### 3.6 Heterogeneity, inconsistency, and sensitivity analysis

#### 3.6.1 Heterogeneity assessment

Potential sources of heterogeneity were systematically assessed through multiple approaches. Clinical heterogeneity was evaluated by examining study characteristics including intervention duration, sample size, intervention type. Statistical heterogeneity was assessed using I² statistics and tau² values for direct comparisons. Although these factors contributed to observed heterogeneity, subgroup analyses revealed that none significantly altered the overall network meta-analysis results or conclusions.

#### 3.6.2 Network inconsistency assessment

Network inconsistency was rigorously evaluated using node-splitting analysis where applicable. For the incidence rate outcome, no closed loops existed in the network structure, thus inconsistency assessment was not applicable. For the effectiveness rate outcome, node-splitting analysis was performed to compare direct and indirect estimates for treatment comparisons with both types of evidence. The results showed no significant inconsistency for any comparison: D-A (P=0.8419), DG-A (P=0.816), G-A (P=0.7317), D-B (P=0.8294), DG-B (P=0.826), and G-B (P=0.7222). All P-values were well above 0.05, indicating good agreement between direct and indirect evidence and supporting the validity of our network meta-analysis assumptions (**Supplementary Figure 2**).

#### 3.6.3 Sensitivity analysis

Sensitivity analyses of 31 studies for primary outcomes demonstrated robust results. Excluding any single study did not materially alter the pooled estimates (incidence rate: OR=0.16, 95%CI [0.11–0.24], P<0.01; effectiveness rate: OR=3.41, 95%CI [1.75–6.65], P<0.01; see **Supplementary Tables 2, 3**). The results remained robust to single-study exclusion, confirming that while high-risk studies may marginally influence effect magnitude, their impact on clinical interpretation is limited (**Supplementary Figures 3, 4**).

### 3.7 Safety and adverse events

Four randomized controlled trials (RCTs) (15, 37, 40, 42) included in this systematic review reported safety outcomes. Three studies documented specific cases of 20 adverse events, while one study reported only statistical data on adverse reaction incidence rates. The safety results for each study are detailed as follows:

Study 1 (40) (Auricular acupoint combined with acupoint application vs. routine care, total sample size n=341).

Intervention group: 6 serious adverse events (SAEs), including chylothorax (n=1), arrhythmia (n=2), deep vein thrombosis (n=1), and hypoxemia (n=1).

Control group: 3 SAEs, including arrhythmia (n=1) and hypoxemia (n=2).

Study 2 (42) (Probiotics vs. placebo, total sample size n=91)

No grade 3 or higher serious adverse events were observed.

The intervention group showed significantly lower incidence of gastrointestinal adverse reactions (nausea, vomiting, diarrhea, anorexia) compared to the control group (p<0.05). No statistically significant differences were found between groups for other adverse events.

Study 3 (15) (combined use of two types of acupoint patches vs. single acupoint application, total sample size n=106)

Intervention group: 5 adverse events, including rash (n=1), diarrhea (n=3), and abdominal pain (n=1).

Control group: 2 adverse events, including diarrhea (n=1) and nausea/vomiting (n=1).

Study 4 (37) (acupoint application vs. placebo, total sample size n=82)

Intervention group: 3 adverse events, including vomiting (n=1) and skin allergy (n=2).

Control group: 2 adverse events, including diarrhea (n=1) and vomiting (n=1).

## 4 Discussion

### 4.1 Methodological quality of included studies

This study included 30 Chinese literature papers and 3 English literature papers, of which 2 were level A evidence, and the remaining 31 were level B evidence. However, there were significant differences in heterogeneity. The English literature had a more rigorous design and relatively higher quality.

### 4.2 Efficacy of NPIs for constipation management in lung cancer patients

Constipation represents one of the prevalent adverse effects encountered during lung cancer treatment protocols, with an exceptionally high incidence among patients undergoing chemotherapy regimens. The clinical presentation of constipation in lung cancer patients typically encompasses a spectrum of manifestations, including defecatory difficulty, reduced stool frequency, and fecal hardening or impaction (43). From a Traditional Chinese Medicine (TCM) perspective, the pathophysiological mechanisms underlying constipation in lung cancer patients are twofold. Primarily, there exists a disruption in the exterior-interior relationship between the lungs and large intestine (known as the “biao-li” connection), resulting in compromised peristaltic function and impaired intestinal transit. Secondarily, deficiencies in vital substances—namely, qi, blood, yin, and yang—coupled with pathological accumulations of phlegm and blood stasis, contribute to dysregulated intestinal motility and aberrant bowel function (44).

#### 4.2.1 Optimal intervention for reducing constipation incidence in lung cancer patients: acupoint stimulation

Our study demonstrates that acupoint stimulation confers significant advantages in reducing constipation incidence amongst lung cancer patients, achieving a noteworthy SUCRA ranking of 77%. These findings Optimal Intervention for Reducing Constipation Incidence in Lung Cancer Patients: Acupoint Stimulation

Our study demonstrates that acupoint stimulation confers significant advantages in reducing constipation incidence amongst lung cancer patients, achieving a noteworthy SUCRA ranking of 77%. These findings correspond with the theoretical framework of “meridian theory” in Traditional Chinese Medicine, which postulates that acupoint stimulation modulates gastrointestinal function via specific energetic pathways. Contemporary biomedical research has elucidated the physiological mechanisms underpinning this therapeutic approach: stimulation of acupoints such as “Zusanli” facilitates activation of the vagal-enteric neural axis, promoting secretion of gastrointestinal peptide hormones and enhancing colonic peristalsis. Research indicates that stimulation of the “Neiguan” and “Zusanli” acupoints significantly reduces the incidence of constipation in lung cancer patients receiving chemotherapy. The underlying mechanism may be associated with inhibiting 5-HT3 receptor hyperactivation and attenuating intestinal water reabsorption (41, 45). Furthermore, acupoint stimulation demonstrates pronounced safety advantages, circumventing adverse effects of pharmacological interventions such as lactulose, including electrolyte disturbances and melanosis coli (46). This therapeutic approach is particularly advantageous for lung cancer patients with concomitant comorbidities or compromised pharmacological tolerance. the theoretical framework of “meridian theory” in Traditional Chinese Medicine, which postulates that acupoint stimulation modulates gastrointestinal function via specific energetic pathways. Contemporary biomedical research has elucidated the physiological mechanisms underpinning this therapeutic approach: stimulation of acupoints such as “Zusanli” facilitates activation of the vagal-enteric neural axis, promoting secretion of gastrointestinal peptide hormones and enhancing colonic peristalsis. Research indicates that stimulation of the “Neiguan” and “Zusanli” acupoints significantly reduces the incidence of constipation in lung cancer patients receiving chemotherapy. The underlying mechanism may be associated with inhibiting 5-HT3 receptor hyperactivation and attenuating intestinal water reabsorption (41, 45). Furthermore, acupoint stimulation demonstrates pronounced safety advantages, circumventing adverse effects of pharmacological interventions such as lactulose, including electrolyte disturbances and melanosis coli (46).This therapeutic approach is particularly advantageous for lung cancer patients with concomitant comorbidities or compromised pharmacological tolerance.

#### 4.2.2 Optimal intervention for enhancing constipation treatment efficacy: two types of acupoint patches combined

This study indicates that combined use of two types of acupoint patches can significantly improve the treatment effect of constipation (SUCRA=100%). Research has confirmed that the combination of compound clove appetizing patch and traditional Chinese medicine acupoint patch can significantly improve the treatment effect of constipation in lung cancer patients. The underlying mechanism involves multi-pathway regulation: acupoint patches facilitate transdermal absorption of pharmacologically active constituents (such as rhubarb and borneol), directly stimulating the enteric neural plexus, while clove constituents ameliorate nausea and enhance gastrointestinal motility (15). However, the anomalously elevated SUCRA value may stem from zero-event bias attributable to the limited sample size (intervention cohort comprising merely 53 subjects, all demonstrating symptomatic improvement). Validation necessitates expanded cohort dimensions, incorporating objective parameters such as fecal short-chain fatty acid concentrations and implementing standardized protocols to establish treatment stability. This modality may be clinically recommended as second-line therapy; however, acupoint selection should be predicated upon syndrome differentiation principles with periodic efficacy assessments.

#### 4.2.3 Most effective overall intervention: acupoint application combined with acupoint massage

The findings of this study demonstrate that the combination of acupoint application and acupoint massage exhibits comprehensive advantages in reducing constipation incidence (SUCRA=67%) and improving treatment efficacy (SUCRA=50%) among lung cancer patients. This combined approach harnesses the synergistic effects of transdermal drug absorption and physical stimulation, offering dual benefits in managing constipation in lung cancer patients. On the one hand, the applied medications (such as rhubarb and borneol) are absorbed through the skin and directly stimulate the intestinal plexus, activating cholinergic neurons in the myenteric plexus of the intestinal wall. This promotes colonic motility while simultaneously regulating gastrointestinal hormone secretion, creating a “local-systemic” dual regulatory effect that effectively prevents constipation (47). On the other hand, acupoint massage (targeting points such as “Tianshu” and “Zhongwan”) activates the “vagus” nerve-gut brain axis through mechanical stimulation, enhancing parasympathetic excitability and improving local microcirculation. This provides energy support for intestinal peristalsis, effectively treating constipation (48). This synergistic “chemical stimulation + physical stimulation” model aligns with the Traditional Chinese Medicine theory of “acupoint synergistic enhancement.” For instance, the combined use of “Tianshu” acupoint (the alarm point of the large intestine) and “Shenque” acupoint (a crucial point on the Conception Vessel) provides direct access to intestinal pathologies, while the pairing of “Zusanli”: (the uniting point of the Stomach meridian) with “Shangjuxu” (the lower uniting point of the Large Intestine) exemplifies the classical principle of “treating internal organs through uniting points.” Modern research further corroborates that combining acupuncture and abdominal massage can significantly reduce colonic transit time, with efficacy superior to acupuncture alone. This suggests that physical stimulation can amplify the regulatory effects of acupuncture therapy.

Furthermore, this combined therapy demonstrates high safety, making it particularly suitable for lung cancer patients with comorbid cardiovascular diseases or poor drug tolerance. Its operational simplicity also enhances patients’ willingness for self-management. In clinical practice, this combined approach is recommended as a core intervention, especially for chemotherapy prevention, patients with poor response or intolerance to laxatives, and long-term intestinal management in elderly or frail patients. Implementation requires a TCM practitioner to select acupoints based on syndrome differentiation, adjust stimulation intensity dynamically according to patient feedback, and assess efficacy and safety regularly.

Although NPIs demonstrate numerical advantages over laxatives in reducing constipation incidence and improving efficacy, some differences did not reach statistical significance. This may be directly related to the mechanism of laxatives (such as polyethylene glycol and lactulose), which rapidly increase intestinal volume through osmotic effects.

However, NPIs offer significant advantages in terms of safety and patient tolerance in the short term. Methods such as acupoint stimulation and massage carry no risk of drug accumulation, demonstrate better patient acceptance, and can simultaneously address comorbidities such as anxiety, aligning with the “bio-psycho-social” medical model. The “Chinese Expert Consensus on Prevention and Treatment of Cancer Drug-Related Nausea and Vomiting (2019 Edition)” also explicitly recommends incorporating NPIs into supportive care for cancer patients to reduce drug-related adverse effects (49).

### 4.3 Advantages, limitations, and outlook

#### 4.3.1 Advantages

1. Safety and Adherence: NPIs (such as acupoint stimulation and massage) carry no risk of drug accumulation and offer superior patient tolerance, making them particularly suitable for individuals with underlying conditions or drug intolerances.
2. Comprehensive Benefits: These interventions can simultaneously improve comorbidities such as anxiety and fatigue (e.g., reduction in SAS scores, SMD=-2.41), aligning with the “bio-psycho-social” medical model. They have been recommended in the “Chinese Expert Consensus on Prevention and Treatment of Cancer Drug-Related Nausea and Vomiting (2019 Edition)”.
3. Robustness of Efficacy: Sensitivity analysis indicates that excluding any single study does not alter the pooled results, suggesting high credibility of the findings.

#### 4.3.2 Limitations

1. Sample Size and Intervention Diversity: The included studies had relatively small sample sizes and diverse intervention types, limiting the generalizability and interpretability of results.
2. Significant Heterogeneity: Intervention duration, sample size, and intervention type (e.g., single vs. combined interventions) represented the primary sources of heterogeneity, potentially affecting result interpretation. Furthermore, the scope of interventions examined in this study was limited and did not encompass all potential non-pharmacological methods. Critically, due to the absence of detailed reporting on lung cancer histological subtypes, TNM staging, and constipation severity in the included studies, we were unable to conduct subgroup analyses to explore their impact—this represented a significant additional contributor to the observed heterogeneity.
3. Quality of Evidence: Most included studies were graded as level B. The CINeMA tool assessment indicated relatively low evidence grades for most comparisons between NPIs, suggesting limited reliability (Details can be found in **Supplementary Tables 2**, **3**).
4. Short-term Focus: Existing research predominantly focuses on short-term efficacy observations, lacking long-term effect assessments, while the long-term efficacy of specific interventions remains unclear.
5. Cultural Context and Generalizability: A significant proportion of included studies were conducted in East Asian countries where traditional Chinese medicine (TCM) interventions are commonly integrated with conventional care. The cultural acceptance, implementation mechanisms, and effectiveness of these TCM-based approaches may differ substantially in Western healthcare settings, where such practices face different regulatory frameworks, professional acceptance, and patient expectations. This cultural specificity may limit the direct applicability of findings to diverse global healthcare contexts.

#### 4.3.3 Future directions

Future research should expand sample sizes and increase the number of studies to enhance the persuasiveness of evidence. Additionally, intervention design and implementation should be further optimized, with standardized operational protocols established to reduce the impact of heterogeneity on research outcomes. Moreover, broadening the scope of NPIs to explore more comprehensive treatment options while strengthening long-term efficacy and cost-effectiveness assessments will help provide more thorough and meaningful treatment recommendations for clinicians and patients.

## 5 Conclusion

This systematic review confirms that combined acupoint application and massage significantly reduce constipation incidence and improve symptoms in lung cancer patients, with superior efficacy compared to single interventions, demonstrating short-term clinical value.

However, critical limitations exist: included studies had extremely short follow-up periods (median ≤1 month), small sample sizes, and complete absence of long-term efficacy and safety data, severely restricting clinical generalizability. Therefore, current results support these interventions only as short-term adjuncts to pharmacological therapy. Clinical practice should involve: carefully developing individualized protocols that prioritize measures with established long-term safety, and avoiding extrapolation of short-term benefits to long-term management while monitoring for delayed adverse effects.

Future research requires large-scale randomized controlled trials (with ≥5 years follow-up), enhanced methodology (blinding, standardized outcomes), and health economic evaluations to validate long-term effectiveness and cost-effectiveness, ultimately providing high-quality evidence for personalized constipation management in lung cancer patients.

## Data Availability

The original contributions presented in the study are included in the article/**Supplementary Material**. Further inquiries can be directed to the corresponding author.
