# Supplementary material for: The efficacy of non-pharmacological interventions for treating constipation symptoms in lung cancer patients: a systematic review and network meta-analysis
Source: Front Oncol. 2025 Aug 18;15:1633167. doi: 10.3389/fonc.2025.1633167 (PMC12399620; doi:10.3389/fonc.2025.1633167)
Supplement: Supplementary file 1 [file DataSheet1.docx]

Supplementary Material

# Supplementary Figures and Tables

**Search strategy**

**A combined subject and free word search was conducted using the following Chinese and English databases: China National Knowledge Infrastructure (CNKI), Wanfang Database, VIP Database, SinoMed, Embase, PubMed, Web of Science, and Cochrane Library. Taking PubMed as an example:**

| **PubMed** **(from the inception to January 16, 2025), n=57** | |
| --- | --- |
| #1 | “Lung Neoplasms”[MeSH Terms] |
| #2 | “Neoplasms, Pulmonary”[Title/Abstract] OR “Neoplasm, Pulmonary”[Title/Abstract] OR “Pulmonary Neoplasm”[Title/Abstract] OR “Pulmonary Neoplasms”[Title/Abstract] OR “Neoplasms, Lung”[Title/Abstract] OR “Lung Neoplasm”[Title/Abstract] OR “Neoplasm, Lung”[Title/Abstract] OR “Lung Cancer”[Title/Abstract] OR “Cancer, Lung”[Title/Abstract] OR “Cancers, Lung”[Title/Abstract] OR “Lung Cancers”[Title/Abstract] OR “Cancer of Lung” OR “Pulmonary Cancer”[Title/Abstract] OR “Cancer, Pulmonary”[Title/Abstract] OR “Cancers, Pulmonary”[Title/Abstract] OR “Pulmonary Cancers”[Title/Abstract] OR “Cancer of the Lung”[Title/Abstract] |
| #3 | #1OR#2 |
| #4 | “Constipation”[Title/Abstract] OR “Colonic Inertia”[Title/Abstract] OR “Dyschezia” [Title/Abstract] |
| #5 | #3AND#4 |

Supplementary Document Table 2: Quality Evaluation of Occurrence Rate Evidence Evaluation (CINeMA)

| Comparison | Number of studies | Within-study bias | Reporting bias | Indirectness | Imprecision | Heterogeneity | Incoherence | Confidence rating |
| --- | --- | --- | --- | --- | --- | --- | --- | --- |
| A:CD | 1 | No concerns | Low risk | Some concerns | No concerns | Major concerns | Major concerns | Low |
| A:D | 3 | Some concerns | Low risk | No concerns | No concerns | Major concerns | Major concerns | Low |
| A:DJ | 1 | No concerns | Low risk | Some concerns | Major concerns | No concerns | Major concerns | Low |
| A:E | 1 | No concerns | Low risk | No concerns | Major concerns | No concerns | Major concerns | Moderate |
| A:G | 2 | Some concerns | Low risk | No concerns | No concerns | Major concerns | Major concerns | Low |
| A:GB | 1 | No concerns | Low risk | Some concerns | Major concerns | No concerns | Major concerns | Low |
| A:GD | 1 | No concerns | Low risk | Some concerns | Major concerns | No concerns | Major concerns | Low |
| A:H | 2 | No concerns | Low risk | No concerns | No concerns | Major concerns | Major concerns | Moderate |
| A:K | 2 | No concerns | Low risk | No concerns | No concerns | Major concerns | Major concerns | Moderate |
| B:D | 1 | No concerns | Low risk | No concerns | No concerns | Major concerns | Major concerns | Moderate |
| B:I | 1 | Major concerns | Low risk | No concerns | Major concerns | No concerns | Major concerns | Low |
| A:B | 0 | Some concerns | Low risk | No concerns | Major concerns | No concerns | Major concerns | Low |
| A:I | 0 | Some concerns | Low risk | No concerns | Major concerns | No concerns | Major concerns | Low |
| B:CD | 0 | No concerns | Low risk | No concerns | No concerns | Major concerns | Major concerns | Moderate |
| B:DJ | 0 | No concerns | Low risk | No concerns | Major concerns | No concerns | Major concerns | Moderate |
| B:E | 0 | No concerns | Low risk | No concerns | No concerns | Major concerns | Major concerns | Moderate |
| B:G | 0 | Some concerns | Low risk | No concerns | No concerns | Major concerns | Major concerns | Low |
| B:GB | 0 | No concerns | Low risk | No concerns | No concerns | Major concerns | Major concerns | Moderate |
| B:GD | 0 | No concerns | Low risk | No concerns | No concerns | Major concerns | Major concerns | Moderate |
| B:H | 0 | Some concerns | Low risk | No concerns | No concerns | Major concerns | Major concerns | Low |
| B:K | 0 | Some concerns | Low risk | No concerns | No concerns | Major concerns | Major concerns | Low |
| CD:D | 0 | Some concerns | Low risk | Some concerns | Major concerns | No concerns | Major concerns | Low |
| CD:DJ | 0 | No concerns | Low risk | Some concerns | Major concerns | No concerns | Major concerns | Low |
| CD:E | 0 | No concerns | Low risk | Some concerns | Major concerns | No concerns | Major concerns | Low |
| CD:G | 0 | Some concerns | Low risk | Some concerns | Major concerns | No concerns | Major concerns | Low |
| CD:GB | 0 | No concerns | Low risk | Some concerns | Major concerns | No concerns | Major concerns | Low |
| CD:GD | 0 | No concerns | Low risk | Some concerns | Major concerns | No concerns | Major concerns | Low |
| CD:H | 0 | No concerns | Low risk | Some concerns | Major concerns | No concerns | Major concerns | Low |
| CD:I | 0 | Some concerns | Low risk | No concerns | Major concerns | No concerns | Major concerns | Low |
| CD:K | 0 | No concerns | Low risk | Some concerns | Major concerns | No concerns | Major concerns | Low |
| D:DJ | 0 | Some concerns | Low risk | Some concerns | Major concerns | No concerns | Major concerns | Low |
| D:E | 0 | Some concerns | Low risk | No concerns | Major concerns | No concerns | Major concerns | Low |
| D:G | 0 | Some concerns | Low risk | No concerns | Major concerns | No concerns | Major concerns | Low |
| D:GB | 0 | Some concerns | Low risk | Some concerns | Major concerns | No concerns | Major concerns | Low |
| D:GD | 0 | Some concerns | Low risk | Some concerns | Major concerns | No concerns | Major concerns | Low |
| D:H | 0 | Some concerns | Low risk | No concerns | Major concerns | No concerns | Major concerns | Low |
| D:I | 0 | Some concerns | Low risk | No concerns | Major concerns | No concerns | Major concerns | Low |
| D:K | 0 | Some concerns | Low risk | No concerns | Major concerns | No concerns | Major concerns | Low |
| DJ:E | 0 | No concerns | Low risk | Some concerns | Major concerns | No concerns | Major concerns | Low |
| DJ:G | 0 | Some concerns | Low risk | Some concerns | Major concerns | No concerns | Major concerns | Low |
| DJ:GB | 0 | No concerns | Low risk | Some concerns | Major concerns | No concerns | Major concerns | Low |
| DJ:GD | 0 | No concerns | Low risk | Some concerns | Major concerns | No concerns | Major concerns | Low |
| DJ:H | 0 | No concerns | Low risk | Some concerns | Major concerns | No concerns | Major concerns | Low |
| DJ:I | 0 | Some concerns | Low risk | No concerns | Major concerns | No concerns | Major concerns | Low |
| DJ:K | 0 | No concerns | Low risk | Some concerns | Major concerns | No concerns | Major concerns | Low |
| E:G | 0 | Some concerns | Low risk | No concerns | Major concerns | No concerns | Major concerns | Low |
| E:GB | 0 | No concerns | Low risk | Some concerns | Major concerns | No concerns | Major concerns | Low |
| E:GD | 0 | No concerns | Low risk | Some concerns | Major concerns | No concerns | Major concerns | Low |
| E:H | 0 | No concerns | Low risk | No concerns | Major concerns | No concerns | Major concerns | Low |
| E:I | 0 | Some concerns | Low risk | No concerns | Major concerns | No concerns | Major concerns | Low |
| E:K | 0 | No concerns | Low risk | No concerns | Major concerns | No concerns | Major concerns | Low |
| G:GB | 0 | Some concerns | Low risk | Some concerns | Major concerns | No concerns | Major concerns | Low |
| G:GD | 0 | Some concerns | Low risk | Some concerns | Major concerns | No concerns | Major concerns | Low |
| G:H | 0 | Some concerns | Low risk | No concerns | Major concerns | No concerns | Major concerns | Low |
| G:I | 0 | Some concerns | Low risk | No concerns | Major concerns | No concerns | Major concerns | Low |
| G:K | 0 | Some concerns | Low risk | No concerns | Major concerns | No concerns | Major concerns | Low |
| GB:GD | 0 | No concerns | Low risk | Some concerns | Major concerns | No concerns | Major concerns | Low |
| GB:H | 0 | No concerns | Low risk | Some concerns | Major concerns | No concerns | Major concerns | Low |
| GB:I | 0 | Some concerns | Low risk | No concerns | Major concerns | No concerns | Major concerns | Low |
| GB:K | 0 | No concerns | Low risk | Some concerns | Major concerns | No concerns | Major concerns | Low |
| GD:H | 0 | No concerns | Low risk | Some concerns | Major concerns | No concerns | Major concerns | Low |
| GD:I | 0 | Some concerns | Low risk | No concerns | Major concerns | No concerns | Major concerns | Low |
| GD:K | 0 | No concerns | Low risk | Some concerns | Major concerns | No concerns | Major concerns | Low |
| H:I | 0 | Some concerns | Low risk | No concerns | Major concerns | No concerns | Major concerns | Low |
| H:K | 0 | No concerns | Low risk | No concerns | Major concerns | No concerns | Major concerns | Moderate |
| I:K | 0 | Some concerns | Low risk | No concerns | Major concerns | No concerns | Major concerns | Low |

Supplementary Document Table 3 Effective Rate Evidence Evaluation Quality Evaluation (CINeMA)

| Comparison | Number of studies | Within-study bias | Reporting bias | Indirectness | Imprecision | Heterogeneity | Incoherence | Confidence rating |
| --- | --- | --- | --- | --- | --- | --- | --- | --- |
| A:D | 3 | No concerns | Low risk | No concerns | No concerns | Major concerns | No concerns | Moderate |
| A:DG | 1 | Some concerns | Low risk | Some concerns | Major concerns | No concerns | No concerns | Low |
| A:G | 3 | Some concerns | Low risk | No concerns | Major concerns | No concerns | No concerns | Low |
| A:GH | 1 | No concerns | Low risk | Some concerns | Major concerns | No concerns | Major concerns | Low |
| A:H | 1 | No concerns | Low risk | Some concerns | Major concerns | No concerns | No concerns | Low |
| B:D | 2 | Some concerns | Low risk | No concerns | Major concerns | No concerns | No concerns | Low |
| B:DG | 1 | Some concerns | Low risk | Some concerns | Major concerns | No concerns | No concerns | Low |
| B:DK | 1 | No concerns | Low risk | Some concerns | Major concerns | No concerns | Some concerns | Low |
| B:G | 2 | Some concerns | Low risk | No concerns | Major concerns | No concerns | No concerns | Low |
| B:HI | 1 | No concerns | Low risk | Some concerns | Major concerns | No concerns | Some concerns | Low |
| D:F | 1 | Some concerns | Low risk | Some concerns | Major concerns | No concerns | Some concerns | Low |
| G:GH | 1 | No concerns | Low risk | Some concerns | Major concerns | No concerns | No concerns | Low |
| G:H | 1 | No concerns | Low risk | Some concerns | Major concerns | No concerns | No concerns | Low |
| GH:H | 1 | No concerns | Low risk | Some concerns | Major concerns | No concerns | Some concerns | Low |
| A:B | 0 | Some concerns | Low risk | No concerns | Major concerns | No concerns | Some concerns | Low |
| A:DK | 0 | Some concerns | Low risk | Some concerns | Major concerns | No concerns | Some concerns | Low |
| A:F | 0 | Some concerns | Low risk | Some concerns | Major concerns | No concerns | Some concerns | Low |
| A:HI | 0 | Some concerns | Low risk | Some concerns | Major concerns | No concerns | Some concerns | Low |
| B:F | 0 | Some concerns | Low risk | Some concerns | Major concerns | No concerns | Some concerns | Low |
| B:GH | 0 | No concerns | Low risk | Some concerns | Major concerns | No concerns | Some concerns | Low |
| B:H | 0 | No concerns | Low risk | Some concerns | Major concerns | No concerns | Some concerns | Low |
| D:DG | 0 | Some concerns | Low risk | No concerns | Major concerns | No concerns | Some concerns | Low |
| D:DK | 0 | Some concerns | Low risk | Some concerns | Major concerns | No concerns | Some concerns | Low |
| D:G | 0 | Some concerns | Low risk | No concerns | Major concerns | No concerns | Some concerns | Low |
| D:GH | 0 | No concerns | Low risk | No concerns | Major concerns | No concerns | Some concerns | Moderate |
| D:H | 0 | No concerns | Low risk | No concerns | Major concerns | No concerns | Some concerns | Moderate |
| D:HI | 0 | Some concerns | Low risk | Some concerns | Major concerns | No concerns | Some concerns | Low |
| DG:DK | 0 | No concerns | Low risk | Some concerns | Major concerns | No concerns | Some concerns | Low |
| DG:F | 0 | Some concerns | Low risk | Some concerns | Major concerns | No concerns | Some concerns | Low |
| DG:G | 0 | Some concerns | Low risk | Some concerns | Major concerns | No concerns | Some concerns | Low |
| DG:GH | 0 | Some concerns | Low risk | Some concerns | Major concerns | No concerns | Some concerns | Low |
| DG:H | 0 | Some concerns | Low risk | Some concerns | Major concerns | No concerns | Some concerns | Low |
| DG:HI | 0 | No concerns | Low risk | Some concerns | Major concerns | No concerns | Some concerns | Low |
| DK:F | 0 | Some concerns | Low risk | Some concerns | Major concerns | No concerns | Some concerns | Low |
| DK:G | 0 | No concerns | Low risk | Some concerns | Major concerns | No concerns | Some concerns | Low |
| DK:GH | 0 | No concerns | Low risk | Some concerns | Major concerns | No concerns | Some concerns | Low |
| DK:H | 0 | No concerns | Low risk | Some concerns | Major concerns | No concerns | Some concerns | Low |
| DK:HI | 0 | No concerns | Low risk | Some concerns | Major concerns | No concerns | Some concerns | Low |
| F:G | 0 | Some concerns | Low risk | No concerns | Major concerns | No concerns | Some concerns | Low |
| F:GH | 0 | Some concerns | Low risk | Some concerns | Major concerns | No concerns | Some concerns | Low |
| F:H | 0 | Some concerns | Low risk | Some concerns | Major concerns | No concerns | Some concerns | Low |
| F:HI | 0 | Some concerns | Low risk | Some concerns | Major concerns | No concerns | Some concerns | Low |
| G:HI | 0 | No concerns | Low risk | Some concerns | Major concerns | No concerns | Some concerns | Low |
| GH:HI | 0 | No concerns | Low risk | Some concerns | Major concerns | No concerns | Some concerns | Low |
| H:HI | 0 | No concerns | Low risk | Some concerns | Major concerns | No concerns | Some concerns | Low |

## Supplementary Figures





**Supplementary Figure 1. Detailed image of literature quality evaluation**





**Supplementary Figure 2. Inconsistency test of efficiency rate**


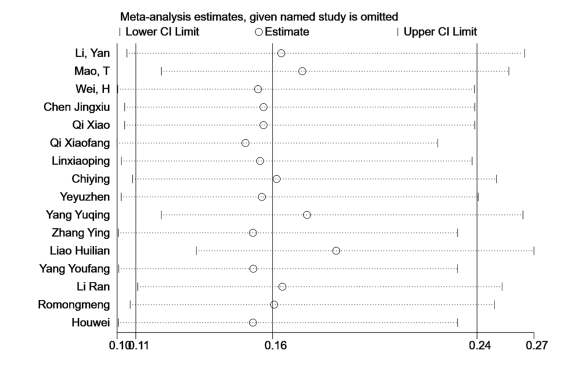


**Supplementary Figure 3. Sensitivity analysis of incidence rate**


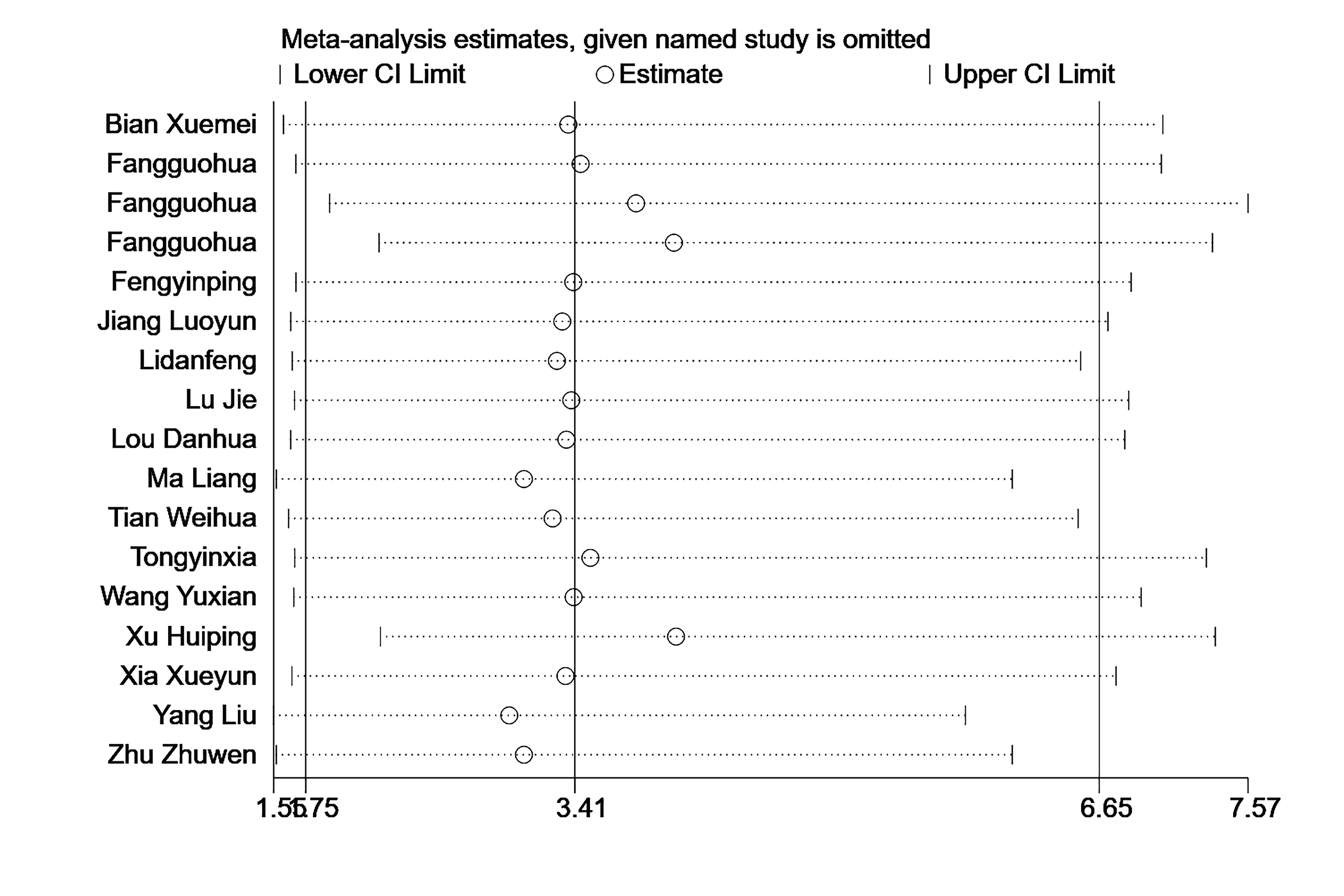


**Supplementary Figure 4. Sensitivity analysis of effective rate**
